# Intramolecular interaction kinetically regulates fibril formation by human and mouse α-synuclein

**DOI:** 10.1038/s41598-023-38070-4

**Published:** 2023-07-05

**Authors:** Takashi Ohgita, Hiroki Kono, Izumi Morita, Hiroyuki Oyama, Toshinori Shimanouchi, Norihiro Kobayashi, Hiroyuki Saito

**Affiliations:** 1 Laboratory of Biophysical Chemistry, Kyoto Pharmaceutical University, 5 Misasagi-Nakauchi-cho, Yamashina-ku, Kyoto, 607-8414 Japan; 2 Department of Bioanalytical Chemistry, Kobe Pharmaceutical University, 4-19-1 Motoyama-Kitamachi, Higashinada-ku, Kobe, 658-8558 Japan; 3 Graduate School of Environmental and Life Science, Okayama University, Okayama, 700-8530 Japan

**Keywords:** Intrinsically disordered proteins, Protein aggregation

## Abstract

Regulation of α-synuclein (αS) fibril formation is a potent therapeutic strategy for αS-related neurodegenerative disorders. αS, an intrinsically disordered 140-residue intraneural protein, comprises positively charged N-terminal, hydrophobic non-amyloid β component (NAC), and negatively charged C-terminal regions. Although mouse and human αS share 95% sequence identity, mouse αS forms amyloid fibrils faster than human αS. To evaluate the kinetic regulation of αS fibrillation, we examined the effects of mismatched residues in human and mouse αS on fibril formation and intramolecular interactions. Thioflavin T fluorescence assay using domain-swapped or C-terminal-truncated αS variants revealed that mouse αS exhibited higher nucleation and fibril elongation than human αS. In mouse αS, S87N substitution in the NAC region rather than A53T substitution is dominant for enhanced fibril formation. Fӧrester resonance energy transfer analysis demonstrated that the intramolecular interaction of the C-terminal region with the N-terminal and NAC regions observed in human αS is perturbed in mouse αS. In mouse αS, S87N substitution is responsible for the perturbed interaction. These results indicate that the interaction of the C-terminal region with the N-terminal and NAC regions suppresses αS fibril formation and that the human-to-mouse S87N substitution in the NAC region accelerates αS fibril formation by perturbing intramolecular interaction.

## Introduction

Aggregation of α-synuclein (αS) into amyloid fibrils in the brains is strongly associated with several neurodegenerative disorders, such as Parkinson’s disease (PD), multiple system atrophy, and dementia with Lewy bodies^1^. Pharmacological inhibition of αS fibril formation is a potent therapeutic strategy for these neurodegenerative diseases. Recent studies have suggested that intrinsically disordered proteins, such as αS^2^ harbor hotspot residues that markedly change the protein conformation^3^. Indeed, some point mutations, such as A30P, E46K, and A53T, which are known risk factors for familial PD, markedly change the conformation of αS, leading to alterations in the fibril-forming propensity of αS^4–11^. Thus, the identification of hotspot residues that determine fibril-forming propensity and the elucidation of the underlying mechanisms will enable the rational design of therapeutic molecules for αS-related neurodegenerative disorders.

**Figure 1:**
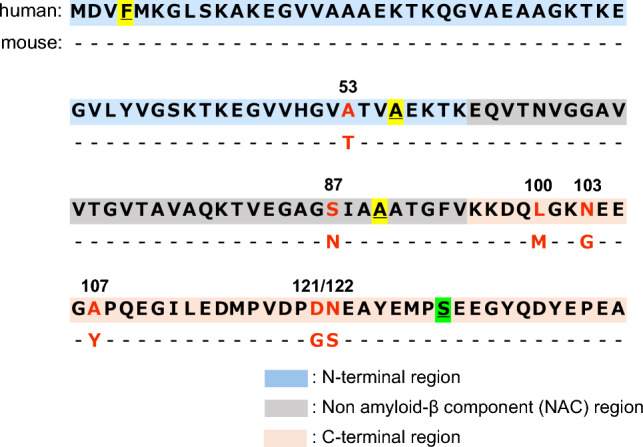
Primary structure alignment of human alpha-synuclein (h-αS) and mouse alpha-synuclein (m-αS). The primary structure of αS comprises the following three regions: the N-terminal (residues 1–60: blue background), a non-amyloid β component (residues 61–95: gray background), and a C-terminal (residues 96–140: orange background). Seven mismatched residues between h-αS and m-αS are represented in red font in the sequence. Replaced Trp and Cys residues are underlined.

αS, an intraneuronal 140-residue protein, comprises a positively charged N-terminal (residues 1–60), a hydrophobic non-amyloid β component (NAC, residues 61–95), and the negatively charged C-terminal (residues 96–140) (Fig. 1)^2,12,13^. In healthy neurons, most of αS exists as a random-coiled monomer^14^, transiently interacting with chaperone proteins (such as Hsp70 and Hsp90)^15^, synaptic vesicles, or plasma membranes^2,13,16–19^. Seven imperfect 11-mer repeat sequences (XKTKEGVXXXX) in the N-terminal and NAC regions form an apolipoprotein-like amphipathic α-helix on lipid membranes^16,17^ and are involved in the regulation of synaptic vesicle clustering and docking^18,19^. Under pathological conditions, αS undergoes a transition to a β-strand-rich structure around the NAC region and aggregates into amyloid fibrils^20–22^. Although the NAC region is necessary and sufficient for the fibril-forming ability of αS^23–25^, the N-terminal and C-terminal regions are reported to kinetically regulate fibril formation^26–31^. Recently, we reported that the PD-related N-terminal A53T mutation enhances the formation of amyloid nuclei (nucleation) by decreasing the enthalpic barrier, whereas the C-terminal truncation promotes autocatalytic amplification of the fibrils^32^. Other factors, such as pH, temperature, and the presence of lipid vesicles, metal ions, and dopamine are reported to induce fibrillation by altering the intramolecular interactions between the N-terminal and C-terminal regions^3,33^. However, the regulatory mechanisms involved in αS fibril formation have not been completely elucidated.

αS is highly conserved in vertebrates. Human αS (h-αS) and mouse αS (m-αS) share 95% sequence identity and exhibit variations in the following seven amino acid residues: A53T in the N-terminal region, S87N in the NAC region, and L100M, N103G, A107Y, D121G, and N122S in the C-terminal region (Fig. 1). Although h-αS and m-αS share high sequence identity, m-αS forms fibrils faster than h-αS^34,35^. Molecular dynamics simulation^36^ and nuclear magnetic resonance analysis^37^ revealed the differences in intramolecular interactions and secondary structural propensity between h-αS and m-αS, indicating the crucial roles of the mismatched residues between h-αS and m-αS in determining tertiary conformations and aggregation propensity. As A53T mutation in h-αS is a risk factor for early-onset PD^38^, m-αS, which harbors an N-terminal T53 residue, exhibits enhanced aggregation in vitro and in vivo^4–6,39^. Although other non-primate αS proteins also share the T53 residue, the fibril-forming propensities of some non-primate αS (e.g., elephant, whale, and pig) proteins are weaker than those of h-αS^40^. This indicates that in addition to the N-terminal T53 residue, other mismatched residues may be involved in the enhanced fibril formation of m-αS. Another feature of the primary structure of m-αS is S87N substitution in the NAC region. The phosphorylation at the S87 residue of h-αS, which is specifically upregulated in the brains of patients with synucleinopathy^41,42^, strongly inhibits αS aggregation by increasing the conformational flexibility of monomers^42,43^. This suggests the potential role of S87N substitution in the regulation of fibril formation. However, the mechanism through which S87N substitution contributes to the rapid fibril formation of m-αS has not been elucidated.

In this study, the fibril-forming properties and intramolecular interactions of h-αS and m-αS variants were comparatively analyzed to understand the mechanism of kinetic regulation of fibril formation by αS. To examine the effects of each mismatched residue on fibril formation, N-terminal-swapped or NAC region-swapped or C-terminal-truncated h-αS and m-αS variants were designed. Fibril formation was traced over time using thioflavin T (ThT), a fibril-specific fluorescent dye, along with oligomer-specific and fibril-specific antibodies. The intramolecular interactions of the αS variants were evaluated by measuring the Förster resonance energy transfer (FRET) from Trp residue introduced in the N-terminus, pre-NAC, or NAC regions to an acrylodan (Ac) molecule attached at the C-terminus. The results of this study indicate that the intramolecular interaction of the C-terminal region with the N-terminal and NAC regions regulates the fibril formation kinetics of αS. The perturbed intramolecular interaction in m-αS accelerates fibril formation.

## Results

### Time traces of fibril formation of h-αS and m-αS

**Figure 2:**
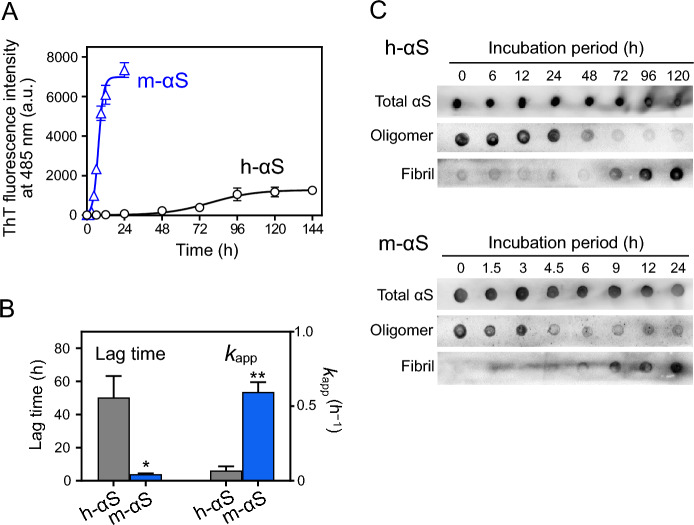
Fibril formation kinetics of human alpha-synuclein (h-αS) and mouse alpha-synuclein (m-αS). (**A**) Thioflavin T (ThT) fluorescence traces for fibril formation of h-αS (○) and m-αS (Δ). αS (100 μM) and 10 μM ThT were incubated at 37 °C with rotation at 30 rpm. The measurements were repeated at least three times using independently prepared samples. *Error bars* represent standard error (S.E.). The *solid lines* represent the curves fitted using the sigmoidal Eq. (1). *a.u.*, arbitrary unit. (**B**) Comparison of lag time and apparent rate constant for fibril growth (*k*_app_) of h-αS and m-αS. *Error bars* represent S.E. *p < 0.05; **p < 0.01 compared with h-αS. (**C**) Dot blot analysis of oligomerization and fibrillation of h-αS (upper panels) and m-αS (lower panels). Total, oligomeric, or fibrillar forms of αS were detected using anti-αS mAb #10–8, anti-oligomer A11 antibody, and anti-fibril mAb#B7-5, respectively. The images of separated blots were individually acquired with different exposure times.

Fibril formation of h-αS and m-αS was traced using the ThT fluorescence assay (Fig. 2A). Consistent with the results of previous reports^34,35^, the increase in ThT fluorescence in m-αS was rapid and high when compared with that in h-αS. Despite the significant difference in ThT intensity, the amounts of fibrils were comparable in both h-αS and m-αS (Supplementary Fig. S1), suggesting that the different ThT responses reflect the structural differences in the resultant fibrils^44^. The shapes of the ThT fluorescence curves of both h-αS and m-αS were sigmoidal with a lag phase. This indicates that fibril formation follows the nucleation-polymerization model, according to which aggregation-competent nuclei are initially formed in the lag phase and subsequently grow into fibrils during the elongation phase^45,46^. The aggregation kinetics of the Gly-Pro-h-αS proteins resembled that of the N-terminally-acetylated h-αS^47,48^, which is the native state of the protein^49^.

By analyzing the ThT fluorescence curves with sigmoidal Eq. (1), the lag time for nucleation and the apparent rate constant for fibril elongation (*k*_app_) were determined (Fig. 2B and Supplementary Table S1). Comparative analysis of the lag times and *k*_app_ values indicated that both nucleation and fibril elongation in m-αS were significantly enhanced when compared with those in h-αS. Additionally, dot blot analysis revealed that both h-αS and m-αS reacted with an oligomer-specific A11 antibody^50^ in the lag phase and subsequently reacted with a fibril-specific monoclonal antibody Ab#B7-5^51^ in the elongation phase (Fig. 2C). This suggested that fibril formation of h-αS and m-αS follows the nucleation-polymerization model.

### Fibril structure and morphology of h-αS and m-αS

**Figure 3:**
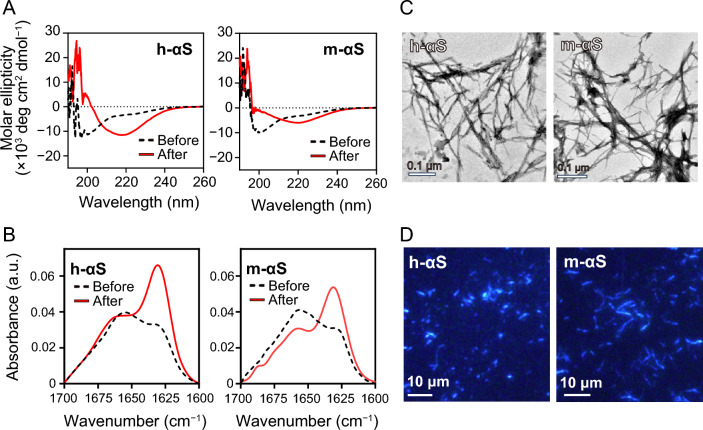
Secondary structure and morphology of human alpha-synuclein (h-αS) and mouse alpha-synuclein (m-αS) fibrils. (**A** and **B**) Changes in the secondary structure of h-αS and m-αS during fibril formation. Far-ultraviolet circular dichroism (CD) (**A**) and attenuated total reflection Fourier-transform infrared (ATR-FTIR) (**B**) spectra before and after incubation at 37 °C for five days. *deg*, degree. *a.u.*, arbitrary units. (**C** and **D**) Transmission electron microscopy (TEM) (**C**) and total internal reflection fluorescence (TIRF) (**D**) images of h-αS and m-αS fibrils. *Scale bars* in* C* and* D* represent 0.1 and 10 μm, respectively.

Far-ultraviolet (UV) circular dichroism (CD) measurements were performed to estimate secondary structural change along with fibril formation (Fig. 3A). Before incubation, the CD spectra of h-αS and m-αS exhibited a single negative peak below 200 nm, which is a characteristic of a random-coil structure. After incubation, the peaks shifted to approximately 220 nm, reflecting the transition to a β-sheet-rich structure during fibril formation. The secondary structure of the fibrils was also evaluated using attenuated total reflection Fourier-transform infrared (ATR-FTIR) measurements (Fig. 3B and Supplementary Table S2). The FTIR spectra of both h-αS and m-αS exhibited an increased band at approximately 1630 cm^–1^ after incubation, indicating a β-sheet-rich structure in the fibrillar state. Transmission electron microscopy (TEM) and total internal reflection fluorescence microscopy (TIRFM) observations of the resultant αS fibrils (Fig. 3C and D, and Supplementary Fig. S2) revealed that both h-αS and m-αS formed thin and straight fibrils. However, the sensitivity of m-αS fibrils to urea denaturation was different from that of h-αS fibrils (Supplementary Fig. S3), and the cross-seeding effects in h-αS and m-αS were much weaker than the self-seedings (Supplementary Fig. S4), suggesting that the detailed structure of fibrils is different between h-αS and m-αS, as previously reported^44^.

### Effect of initial monomer concentration and seed fibrils on αS aggregation kinetics

**Figure 4:**
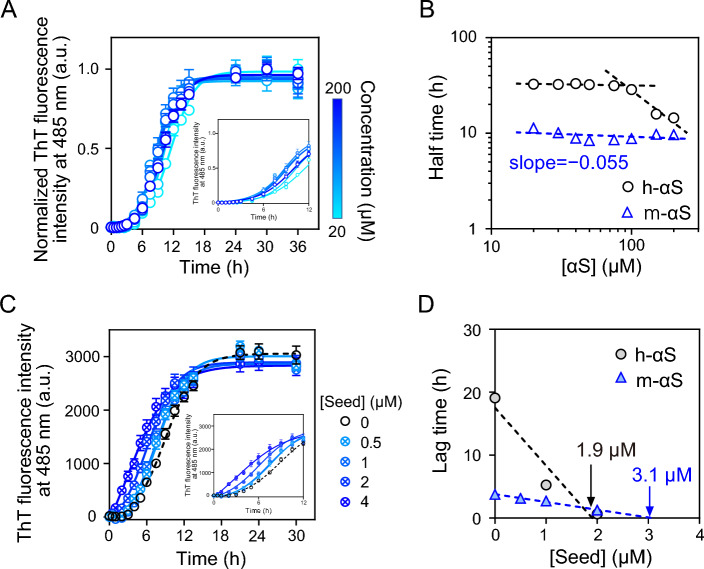
Effect of initial monomer concentration and preformed seed fibril of human alpha-synuclein (h-αS) and mouse alpha-synuclein (m-αS) on fibril formation kinetics. (**A**) Normalized thioflavin T (ThT) fluorescence curves for fibrillation of m-αS at different initial monomer concentrations. Each experiment was repeated at least three times, and three samples were used for each replicate. The *solid lines* represent the curves fitted using sigmoidal Eq. (1). The inset shows the enlarged graphs at the early time points. *Error bars* represent standard error (S.E.). *a.u.*, arbitrary units. (**B**) Double logarithmic plots for half-time for fibril formation vs. initial monomer concentration. Half-time was determined according to sigmoidal Eq. (1). The *dashed lines* represent the linear regression lines. *Error bars* represent S.E. Data for h-αS were obtained from our previous study^33^. (**C**) Traces of ThT fluorescence for fibril formation of m-αS with varying concentrations of preformed seed fibrils. Six samples were used for each replicate, and the experiments were independently repeated three times. The *solid and dotted lines* represent the curves fitted using sigmoidal Eq. (1). The inset shows the enlarged graphs at the early time points. *Error bars* represent S.E. *a.u.*, arbitrary units. (**D**) Correlation of lag time with seed fibril concentration. *Error bars* represent S.E. The *dotted lines* represent the linear regression lines. The data for h-αS are from our previous study^33^.

Next, the dependency of the aggregation kinetics on the initial monomer concentration was examined. Figure 4A shows the normalized ThT fluorescence curves of m-αS at various monomer concentrations. The half-time for fibril formation was defined as the time at which the ThT fluorescence intensity reached 50% of the intensity at the plateau phase. Double logarithmic plots of the half-time and initial monomer concentration (Fig. 4B) enabled the estimation of the dominant step in the aggregation of αS^52,53^. Previously, we demonstrated that the plots of h-αS exhibited a positive curvature at approximately 100 μM, indicating the dominance of surface-catalyzed secondary nucleation at higher concentration^32,53^. In contrast, this curvature was not observed in the half-time plot of m-αS, which had a slope close to 0 in the concentration range of 20–200 μM. This suggests that primary nucleation followed by saturated elongation or saturated secondary nucleation is dominant in the fibril formation of m-αS^52,53^.

The effects of seed fibrils on the aggregation kinetics of m-αS were also examined. Figure 4C shows the ThT fluorescence curves of m-αS in the presence of different concentrations of preformed seed fibrils. The lag time decreased with increasing concentration of seed fibrils and was eliminated at a seed concentration of 4 μM. The seeding effect was more apparently observed under quiescent conditions, in which m-αS did not form fibrils autonomically (Supplementary Fig. S5). These results indicate that primary nucleation is a dominant process in the lag phase^54^. As shown in Fig. 4D, the lag times of both h-αS and m-αS decreased linearly with increasing seed concentrations. From the x-intercept in Fig. 4D, the maximal concentrations of nuclei formed during the lag phase for h-αS and m-αS were estimated to be 1.9 ± 0.7 and 3.1 ± 0.2 μM, respectively. This suggests that m-αS forms a similar number of nuclei as h-αS despite rapid nucleation.

### Effect of mismatched residues on fibril formation of h-αS and m-αS

**Figure 5:**
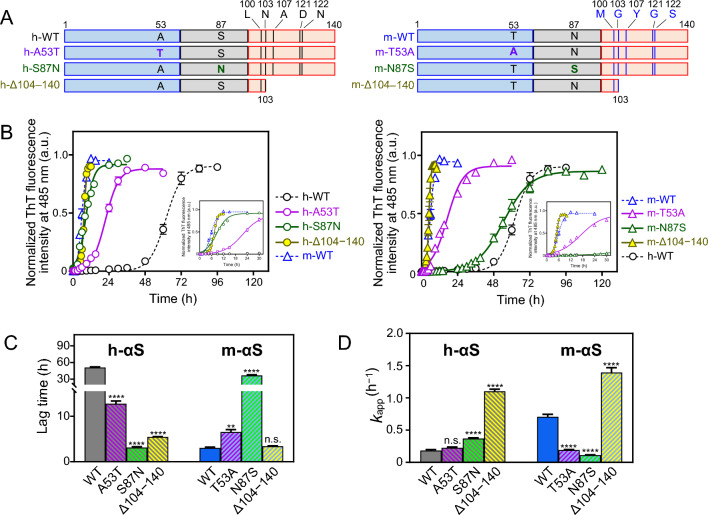
Effects of residue substitution in the N-terminal or non-amyloid β component (NAC) regions and C-terminal truncation on fibril formation of human alpha-synuclein (h-αS) and mouse alpha-synuclein (m-αS). (**A**) Schematic representation of the αS variants used in this assay. The N-terminal, NAC, and C-terminal regions are represented using blue, gray, and orange colors, respectively. (**B**) Normalized thioflavin T (ThT) fluorescence curves for fibril formation of the N-terminal (purple)-substituted or NAC (green)-substituted h-αS (left) and m-αS (right) variants. αS (50 μM) and 10 μM ThT were incubated at 37 °C in 96-well plates with shaking at 500 rpm. Five samples were used for each replicate, and the experiments were independently repeated three times. The insets show the enlarged graphs at the early time points. *Error bars* represent standard error (S.E.). The *solid and dotted lines* represent the curves fitted using the sigmoidal Eq. (1). *a.u.*, arbitrary unit. (**C** and **D**) Lag time and apparent rate constant for fibril growth, *k*_app_ of h-αS and m-αS variants obtained from the curve fitting of the ThT traces shown in Fig. 5B with sigmoidal Eq. (1). *Error bars* represent S.E. **p < 0.01; ****p < 0.0001 compared with the wild-type. n.s., not significant.

To evaluate the effects of each mismatched residues on the fibril formation, the kinetic behavior of ThT fluorescence increases was compared between the N-terminal-swapped or NAC region-swapped or the C-terminal-truncated h-αS and m-αS variants (Fig. 5B). The assay was performed with agitation because no increase in ThT fluorescence was observed in all the αS variants under quiescent conditions (Supplementary Fig. S6). In h-αS variants, all modifications accelerated the increase in ThT fluorescence in the following order: Δ104–140 > S87N > A53T > wild-type (WT). The ThT fluorescence curves of the h-αS S87N and Δ104–140 variants overlapped with that of m-αS WT, indicating that the NAC S87 residue and the C-terminal region are mainly responsible for the suppression of fibril formation in h-αS. In contrast, the T53A and N87S mutations in m-αS variants markedly inhibited fibril formation. The ThT fluorescence increase of the N87S variant was similarly to that of h-αS WT. However, the C-terminal truncation did not affect the fibril formation of m-αS.

The kinetic parameters shown in Fig. 5C revealed that a single substitution at the eighty-seventh residue in the NAC region markedly changed the lag time of h-αS and m-αS. The lag time of the h-αS S87N variant was similar to that of WT m-αS. Similarly, the lag time of the m-αS N87S variant was similar to that of WT h-αS. Additionally, 104–140 truncation in h-αS significantly decreased the lag time, which was similar to that of WT m-αS. These results indicate that the C-terminal region suppresses the nucleation step of h-αS and that S87N substitution in h-αS disrupts the suppression by the C-terminal region, resulting in rapid nucleation, similar to m-αS. Although substitution of the N-terminal fifty-third residue affected the lag times of h-αS and m-αS, the effects were weaker than those observed with the eighty-seventh residue substitution.

C-terminal 104–140 residue truncation also significantly affected the *k*_app_ values of both h-αS and m-αS (Fig. 5D), indicating that the C-terminal region suppresses fibril elongation in h-αS and m-αS. In contrast, the effects of the fifty-third or eighty-seventh residue substitutions on the *k*_app_ values were moderate in both h-αS and m-αS, suggesting the involvement of other substitutions into the regulation of fibril elongation.

### Thermodynamic analysis of fibril formation by h-αS and m-αS

**Figure 6:**
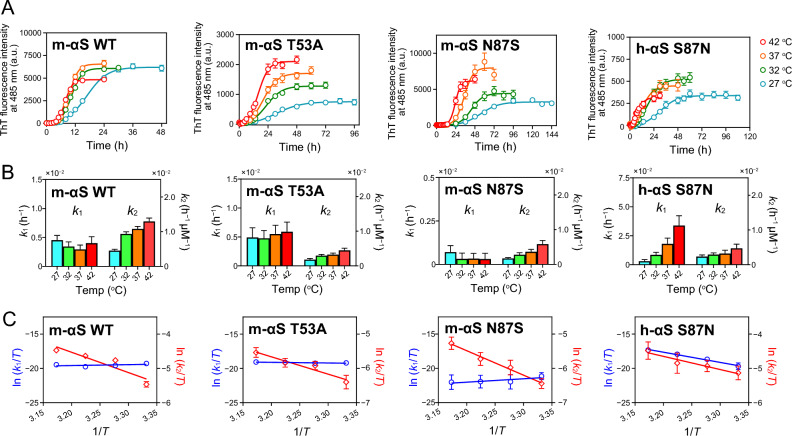
Effect of temperature on the fibril formation kinetics of human alpha-synuclein (h-αS) and mouse alpha-synuclein (m-αS) variants. (**A**) Thioflavin T (ThT) fluorescence curves for fibril formation of h-αS and m-αS variants at different temperatures. Five samples were used for each replicate, and the measurements were independently repeated three times. *Error bars* represent standard error (S.E.). The *solid lines* are the curves fitted using the Finke–Watzky 2-step model. *a.u.*, arbitrary unit. (**B**) Temperature dependence of rate constants for nucleation (*k*_1_) and fibril growth (*k*_2_) for fibril formation of h-αS and m-αS variants. The rate constants were determined according to the Finke–Watzky 2-step model. *Error bars* represent S.E. (**C**) Eyring plots of *k*_1_ (blue) and *k*_2_ (red) for fibril formation. *Error bars* represent S.E. The *solid lines* represent the linear regression lines.

To understand the thermodynamic characteristics of fibril formation by h-αS and m-αS, the temperature dependence of the fibril formation kinetics of m-αS variants was compared with that of their h-αS counterparts^32^. Figure 6A shows the change in the ThT fluorescence curves for fibril formation by h-αS and m-αS variants at different temperatures. Based on the Finke–Watzky kinetic model of homogeneous nucleation, followed by autocatalytic heterogeneous fibril growth^55,56^, the rate constants *k*_1_ for nucleation and *k*_2_ for fibril growth were obtained at each temperature (Fig. 6B). In the m-αS variants, *k*_1_ values were weakly dependent on temperature, whereas *k*_2_ values increased with increasing temperature. From the Eyring plots for the *k*_1_ and *k*_2_ values, the thermodynamic parameters for nucleation and fibril growth were obtained (Fig. 6C). Table 1 summarizes the activation enthalpy Δ*H*^‡^, the activation entropy Δ*S*^‡^, and the activation Gibbs free energy Δ*G*^‡^ for nucleation and fibril growth.

**Table 1:** Thermodynamic parameters for nucleation and fibril growth of human alpha-synuclein (h-αS) and mouse alpha-synuclein (m-αS) variants.

	Nucleation, *k*_1_			Fibril growth, *k*_2_		
	Δ*H*^‡^ (kJ mol^−1^)	*T*Δ*S*^‡^ at 37 °C (kJ mol^−1^)	Δ*G*^‡^ at 37 °C (kJ mol^−1^)	Δ*H*^‡^ (kJ mol^−1^)	*T*Δ*S*^‡^ at 37 °C (kJ mol^−1^)	Δ* G*^‡^ at 37 °C (kJ mol^−1^)
h-αS WT	87 ± 9	− 26 ± 9	113	59 ± 16	− 17 ± 16	76
h-αS A53T	52 ± 8	− 56 ± 8	108	53 ± 12	− 22 ± 11	75
h-αS S87N	124 ± 9	16 ± 9	108	32 ± 6	− 44 ± 6	76
m-αS WT	− 11 ± 15	− 123 ± 15	112	49 ± 15	− 24 ± 15	73
m-αS T53A	8 ± 4	− 102 ± 4	111	42 ± 8	− 34 ± 8	76
m-αS N87S	− 41 ± 21	− 159 ± 21	118	59 ± 5	− 17 ± 5	76

The values of Δ*H*^‡^ and Δ*S*^‡^ were determined from the slope and y-intercept of Eyring plots (Fig. 6C). The values of Δ*G*^‡^ were calculated from the Δ*H*^‡^ and Δ*S*^‡^ values using the equation Δ*G*^‡^ = Δ*H*^‡^ − *T*Δ*S*^‡^ at 37 °C. Data for the h-αS WT and A53T variants were obtained from our previous study^33^. WT, wild-type; m-αS, mouse alpha-synuclein; h-αS, human alpha-synuclein.

In h-αS variants, the free energy barrier for nucleation largely originated from large positive activation enthalpy values^32^. However, in the m-αS variants, the activation enthalpy values for nucleation were close to zero or negative, indicating that the free energy barrier for nucleation is almost entirely entropic. In the h-αS S87N variant, a large increase in the unfavorable activation enthalpy and the concomitant favorable activation entropy for nucleation were observed. However, N87S substitution in m-αS increased the entropic barrier for nucleation. These results indicate that the effects of NAC substitution on the thermodynamic mechanism of nucleation differ between h-αS and m-αS. For fibril growth, large positive activation enthalpy and negative activation entropy values were observed for all h-αS and m-αS variants, indicating that the free energy barrier for fibril growth comprises both enthalpic and entropic barriers.

### FRET analysis to estimate intramolecular interaction in h-αS and m-αS

To examine the regulatory mechanism of fibril formation by h-αS and m-αS mediated by the C-terminal region, FRET analyses were performed to evaluate the intramolecular interactions mediated by the C-terminal region. F4 or A56 in the N-terminal region or A90 in the NAC region was replaced by a Trp residue. The Cys residue for Ac labeling was introduced at the S129 residue in the C-terminal region (Fig. 1). In h-αS, the variants showed accelerated fibril formation, while in m-αS, similarly formed fibrils to WT (Supplementary Fig. S7). The overlapped ThT fluorescence curves in the three h-αS variants suggest the relevance of the S129C substitution on the acceleration in agreement with the previous report^57^.

**Figure 7:**
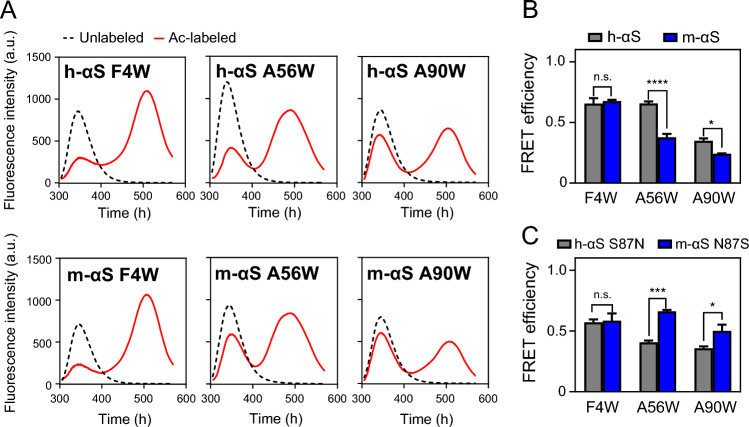
Förster resonance energy transfer (FRET) analysis for intramolecular interaction in human alpha-synuclein (h-αS) and mouse alpha-synuclein (m-αS). (**A**) Fluorescence emission spectra excited at 295 nm for unlabeled (dashed black line) or acrylodan (Ac)-labeled (solid red line) αS S129C variants harboring Trp residues at different sites. The upper and lower panels represent the spectra of h-αS and m-αS variants, respectively. The fluorescence spectra were measured at a protein concentration of 2 μM in phosphate-buffered saline. Each measurement was repeated thrice using independently prepared samples. (**B**) FRET efficiencies for the h-αS and m-αS variants. The value was calculated as 1–*F*_DA_/*F*_D_, where *F*_DA_ and *F*_D_ are the Trp fluorescence intensities of Ac-labeled and unlabeled proteins at 345 nm, respectively. *Error bars* represent standard error (S.E.). *p < 0.05; ****p < 0.0001 compared with the h-αS counterpart. n.s., not significant. (**C**) FRET efficiencies of the S87N h-αS and N87S m-αS variants. *Error bars* represent S.E. *p < 0.05; ***p < 0.001 compared with the h-αS counterpart. n.s., not significant.

Figure 7A shows fluorescence emission spectra of Ac-labeled or unlabeled Trp-introduced variants of h-αS and m-αS. Compared with that in the unlabeled h-αS and m-αS variants, the Trp fluorescence peak significantly decreased at approximately 345 nm with a concomitant appearance of the Ac fluorescence peak at approximately 510 nm in all Ac-labeled variants, indicating the occurrence of FRET from Trp residue to Ac in h-αS and m-αS. These differences in Trp and Ac fluorescence between the unlabeled and Ac-labeled αS variants almost disappeared after complete unfolding in the presence of guanidine hydrochloride (Gdn-HCl) as evidenced by the behaviors of F4W h-αS and m-αS variants (Supplementary Fig. S8). This indicates that FRET behavior reflects the proximity of the C-terminal region with the N-terminal or NAC regions in the folded αS. We have noted minimal effects of the Trp substitutions on the conformational stability of αS^58,59^, as changes in the peak area of Trp fluorescence with increasing concentrations of Gdn-HCl overlapped regardless of the location of the Trp residue (Supplementary Fig. S9). However, differences in the peaks of Ac fluorescence in the Trp-substituted variants (Supplementary Fig. S10) indicate that local hydrophobicity around Ac molecules differed slightly among the variants.

The FRET efficiencies calculated from the differences in Trp fluorescent intensity at 345 nm between Ac-labeled and unlabeled h-αS and m-αS variants are shown in Fig. 7B. In h-αS, the FRET efficiency of the F4W and A56W variants was higher than that of the A90W variant. This indicates that the C-terminal region of h-αS interacts strongly with the N-terminus and the pre-NAC region (residues 47–56)^31,60^ when compared with the NAC region. In m-αS, the FRET efficiency of the A56W and A90W variants was significantly lower than that of h-αS (Fig. 7B). These results indicated that the intramolecular interaction of the C-terminal region with the pre-NAC and NAC regions was perturbed in m-αS.

FRET assays were also performed with the h-αS S87N and m-αS N87S variants to examine the effect of the substitution on the eighty-seventh residue on the intramolecular interaction. The S87N mutation in h-αS markedly decreased the FRET efficiency of the A56W variant, whereas the N87S mutation in m-αS markedly increased the FRET efficiency for the A56W and A90W variants (Fig. 7C and Supplementary Fig. S11). This indicates that the eighty-seventh residue in the NAC region plays a crucial role in regulating the intramolecular interaction of the C-terminal region with the pre-NAC and NAC regions in αS.

## Discussion

αS exists in different conformations owing to its intrinsically disordered characteristic^14^. Thus, alterations in the protein sequence markedly affect the distribution of conformations, contributing to different aggregation propensities of different species. Consistent with previous studies^34,35^, the present study demonstrated that m-αS forms fibrils faster than h-αS even though m-αS and h-αS exhibit a high sequence identity (Fig. 1). This study performed a physicochemical examination of fibril formation and analyzed intramolecular interactions of h-αS and m-αS variants to gain insights into the molecular basis for their different fibril-forming properties. Kinetic analyses revealed that h-αS and m-αS aggregate into fibrils through the nucleation-polymerization mechanism. In this mechanism, both nucleation and fibril elongation are markedly accelerated in m-αS (Fig. 2). Although h-αS and m-αS exhibit large differences in fibril-forming kinetics, the fibrils formed by h-αS and m-αS have similar secondary structures and morphologies (Fig. 3). Analysis of the effects of initial concentrations of monomer or preformed seed fibril concentration on the kinetic parameters indicates that primary nucleation followed by saturated elongation^52,53^ is a dominant process for fibril formation of m-αS. In contrast, surface-catalyzed secondary nucleation was dominant in h-αS at higher concentrations (Fig. 4).

Intramolecular long-range interactions have been reported between different regions of αS^27,30,61^. FRET analyses demonstrated that h-αS exists in a conformation with the C-terminal region located close to the pre-NAC and NAC regions. These intramolecular interactions are largely disrupted in m-αS (Fig. 7). ThT fluorescence and FRET analyses of the residue-substituted variants of h-αS and m-αS indicated that the S87N substitution in the NAC region is mainly relevant for the rapid nucleation and perturbed intramolecular interactions of m-αS (Figs. 5 and 7). This study demonstrated that C-terminal truncation accelerated nucleation in h-αS but not in m-αS (Fig. 5), which indicates the importance of intramolecular interactions between the N-terminal and C-terminal regions in the different nucleation behaviors of h-αS and m-αS. Thus, NAC S87N substitution in m-αS may perturb the intramolecular interactions of the C-terminal region with the pre-NAC and NAC regions, resulting in rapid nucleation and fibril formation. Consistently, the exposure of N-terminal and NAC regions to solvent is directly proportional to the nucleation of αS^30^. The intramolecular N-terminal and C-terminal interactions suppress the solvent exposure of the N-terminal and NAC regions^30,62^. As the A53T mutation in h-αS was reported to destabilize the intramolecular interactions between the N- and C-terminal regions^63,64^, both A53T and S87N substitutions may enhance the nucleation of αS via a common mechanism.

ThT fluorescence analysis (Fig. 5) demonstrated that the S87N substitution is more relevant for accelerating h-αS fibril formation than the A53T substitution. Consistently, S87N substitution had higher effects on the activation enthalpy and entropy for nucleation and fibril growth than A53T substitution (Table 1). However, a previous study reported that the effect of A53T substitution was more significant than that of S87N substitution^34^. This inconsistency might be due to the difference in the experimental conditions of the aggregation assay. The protein concentration used to perform the assay in the previous study (140 μM) was higher than that used in this study (50 μM), which is higher than that in physiological neurons^65^. Previously, we demonstrated that the nucleation of the h-αS A53T variant was markedly enhanced at concentrations above 75 μM^32^. Thus, at high protein concentrations, the effect of S87N substitution might be masked because of the strong effect of the A53T substitution.

In contrast to nucleation, the acceleration of fibril elongation in m-αS cannot be explained only by the perturbation of intramolecular interactions in αS. As fibril elongation occurs through binding of monomers to amyloid nuclei or fibrils^66^, both monomeric and oligomeric or fibrillar states affect fibril elongation propensity. The amide group of glutamine and asparagine sidechains stabilizes the fibril structure via the formation of intermolecular hydrogen bond ladders^21,67–70^. In αS fibrils, the Q79 residue located in the loop of β6-loop-β7 site forms ladders, contributing to the stabilization of fibrils^21^. As the S87 residue is also located in the same loop^21^, the substituted N87 residue in m-αS may form additional hydrogen bond ladders, resulting in stabilized binding of monomers to fibril ends, which enhances fibril elongation. Consistent with this hypothesis, recent studies have reported that the E83Q mutation located in the same loop markedly accelerated fibril formation^71,72^. Substitution at the eighty-seventh residue exerted significant effects on the activation enthalpy of fibril growth in h-αS and m-αS (Table 1), indicating that the eighty-seventh residue in the NAC region is important for the enthalpic regulation of fibril growth.

In summary, this study demonstrated that the interaction of the C-terminal region with the N-terminal and NAC regions suppresses the fibril formation of αS and that the human-to-mouse S87N substitution in the NAC region accelerates αS fibril formation by perturbing intramolecular interactions. Although m-αS aggregates into fibrils faster than h-αS, aged mice do not exhibit neurodegeneration or intracellular aggregation^73^. However, the negligible difference in the toxicity levels between the h-αS and m-αS fibrils does not sufficiently account for this effect (Supplementary Fig. S12). Endogenous m-αS inhibits the formation of Lewy-like inclusions by h-αS in cultured neurons and in mouse brain^74^, which can be attributed to decreased formation of toxic oligomers on the membranes^35^. Further studies are needed to understand the correlation between fibril formation propensity and αS cytotoxicity.

## Methods

### Preparation of recombinant αS proteins

Recombinant h-αS and m-αS variants were prepared as described previously^32,51^. Briefly, the proteins fused to thioredoxin and hexa-histidine tags at N-terminus were expressed in *E. coli* BL21 Star (DE3) and isolated using Ni-affinity chromatography. After cleavage with HRV-3C protease, the fused tags were removed by repeatedly passing through a Ni-chelating column. The resultant proteins possessed two extra amino acids (Gly-Pro) at the N-terminus. The purity of proteins was > 95% as determined using sodium dodecyl sulfate–polyacrylamide gel electrophoresis and Coomassie-blue staining.

### Preparation of αS seed fibrils

The seed fibrils were prepared as described previously^32^. Briefly, the fibrils formed after incubation of 200 μM monomeric αS solution in 96-well plate at 37 °C with shaking were precipitated using ultracentrifugation at 280,000 g for 1.5 h. The supernatant was carefully removed, and the pellet of amyloid fibrils was washed twice with phosphate-buffered saline (PBS) and resuspended in PBS using sonication for 1 min. The resultant amyloid fibrils solution was stored at 4 °C and continuously sonicated for 1 min using a bath-type sonicator (Branson Co., Shelton, CT) before use.

### Generation of anti-αS monoclonal antibody

Four female BALB/c mice (aged 8 weeks; Japan SLC, Hamamatsu, Japan) were immunized with recombinant h-αS protein at approximately two-week intervals. The protein (50 µg/mouse) was subcutaneously injected with Freund's complete adjuvant (primary immunization) or incomplete adjuvant (booster immunizations) emulsified with sterile saline (1:1; 0.2 mL/mouse)^75,76^. On day 7 post-third booster injection, blood samples were collected from individual mice. The titer of serum antibodies against the h-αS protein was determined using the enzyme-linked immunosorbent assay. Mice with the highest titer received intraperitoneal and intrasplenic injections of h-αS protein (50 µg). On day 3 post-administration, splenocytes were fused with P3/NS1/1-Ag4-1 (NS-1) myeloma cells^77^ using a 40% polyethylene glycol 4000 solution^75,76^. The hybridomas secreting desirable antibodies were cloned by limiting dilution, and one of the clones #10-8 was cultured on a large scale. The monoclonal antibody (mAb#10-8) in the resulting culture supernatant was used for subsequent experiments. All experiments on animals were carried out in accordance with guidelines and regulations established in Kobe Pharmaceutical University and all experimental protocols were approved by the Institutional Animal Care and Use Committee. All methods were reported in accordance with the Animal Research: Reporting of in vivo Experiments (ARRIVE) guidelines.

### ThT fluorescence assay

Lyophilized αS proteins suspended in 20 mM glycine buffer (pH 8.0) were solubilized by adding 2 M NaOH^78,79^. To remove the remaining insoluble aggregates, the solution was centrifuged at 10,000 g and 4 °C for 30 min. The supernatant was dialyzed against PBS containing 0.02% NaN_3_ overnight. The solution was centrifuged, and the supernatant was used in the experiments. The western blotting analysis using anti-αS polyclonal antibody (Proteintech, #10842-1-AP) and the dynamic light scattering measurement^80^ did not detect any oligomeric species in the supernatant (Supplementary Fig. S13). Monomeric αS solution supplemented with 10 μM ThT was incubated at 37 °C in a 96-well black plate (Thermo Fisher Scientific, MA, USA) in the presence of Teflon polybeads (1/8″ diameter, ASONE, Osaka, Japan) with shaking at 500 rpm. At each time points, the ThT fluorescence intensity at 485 nm was measured with an excitation wavelength of 440 nm using an Infinite M200 microplate reader (Tecan, Männedorf, Switzerland).

### Kinetic and thermodynamic analyses of fibril formation

The obtained ThT fluorescent curves for fibril formation of αS were analyzed using the sigmoidal Eq. (1)^81,82^ to determine the lag time for nucleation and the *k*_app_ value for fibril growth,

1 $$ F = F_{0} + \frac{{F_{\max } - F_{0} }}{{1 + \exp \left[ {k_{{{\text{app}}}} (t_{{{1 \mathord{\left/ {\vphantom {1 2}} \right. \kern-0pt} 2}}} - t)} \right]}} $$

where *F* is the fluorescence intensity at each time point, *F*_0_ is the initial baseline value during the lag phase and *F*_max_ is the final baseline value after the growth phase. *t*_1/2_ describes the time at which the ThT fluorescence intensity reached 50% of the intensity at the final baseline value. Lag time was calculated as *t*_1/2_ − 2/*k*_app_.

To determine the rate constants *k*_1_ for nucleation and *k*_2_ for fibril growth, the ThT fluorescence curves were also analyzed based on the Finke–Watzky 2-step nucleation-autocatalytic growth model^55,56^,

2 $$ \frac{{F - F_{0} }}{{F_{\max } - F_{0} }} = 1 - \frac{{k_{1} + k_{2} [{\text{A}}]_{0} }}{{k_{1} \exp \left( {k_{1} + k_{2} [{\text{A}}]_{0} } \right)t + k_{2} [{\text{A}}]_{0} }} $$

where [A]_0_ describes the initial monomer concentration.

Thermodynamic parameters for fibril formation were obtained using the Eyring equation^32,83^

3 $$ \ln \left( \frac{k}{T} \right) = - \frac{{\Delta H^{\ddag } }}{R}\frac{1}{T} + \frac{{\Delta S^{\ddag } }}{R} + \ln \left( {\frac{{k_{B} }}{h}} \right) $$

where *k*_B_ and *h* are the Boltzmann’s and Planck’s constants, respectively. The activation enthalpy (Δ*H*^‡^) and entropy (Δ*S*^‡^) were determined from the slope and *y*-intercept of the regression line based on Eq. (3). Δ*G*^‡^ was calculated from Δ*H*^‡^ and Δ*S*^‡^ as Δ*G*^‡^ = Δ*H*^‡^ − *T*Δ*S*^‡^ at 37 °C.

### FRET analysis

Before Ac labeling, a tenfold molar excess of tris (2-carboxyethyl) phosphine hydrochloride (Pierce, Rockford, IL, USA) was incubated with the solution of αS S129C variants for 1 h to reduce the sulfhydryl group. Next, the sample was incubated with Ac (6-acryloyl-2-dimethyaminonaphthalene; Molecular Probes, Inc., Eugene, OR) in dimethyl sulfoxide at a final probe-to-protein ratio of 10:1 (mol/mol) at 25 °C for 3 h in the dark with stirring. The solution was repeatedly dialyzed against PBS (pH7.4) to remove unreacted Ac. The ratio of Ac-labeled protein calculated using an extinction coefficient for Ac of 19,200 M^−1^ at 391 nm was more than 90% for all variants.

The fluorescence spectra of Ac-labeled and unlabeled αS variants (2 μM) were measured using a Hitachi F-7000 fluorescence spectrophotometer at 25 °C. The spectra were recorded from 300 to 600 nm at an excitation wavelength of 295 nm. FRET efficiency was determined using the equation *E* = 1 − *F*_DA_/*F*_D_, where *F*_DA_ and *F*_D_ are the Trp fluorescence intensities of Ac-labeled and unlabeled proteins at 345 nm, respectively. We note that it was previously reported that no intermolecular interactions of αS were observed at our low-concentration condition^31^.

### Dot blot analysis

The monomeric αS solution (100 μM) in a 2-ml protein-low binding tube (Sarstedt, Princeton, NJ) was incubated at 37 °C and 30 rpm. An aliquot of the solution was sampled at each time point and was blotted on a nitrocellulose transfer membrane (0.2 μm pore size; Bio-Rad). The membranes were probed with the anti-αS monoclonal (mAb#10-8), the anti-oligomer A11 polyclonal (Thermo Fisher Scientific, MA, USA), or the anti-fibril monoclonal (mAb#B7-5) antibodies^51^, followed by incubation with the horseradish peroxidase-conjugated secondary antibodies. Immunoreactive signals were developed using an enhanced chemiluminescence prime western blotting detection reagent (GE Healthcare, Milwaukee, WI). The signals were visualized using a LuminoGraph I imaging system (ATTO Corporation, Tokyo, Japan).

### CD spectroscopy

Far-UV CD spectra were measured in the wavelength range 190–260 nm at 25 °C using a Jasco J-1500 spectropolarimeter (JASCO, Tokyo, Japan). h-αS and m-αS solutions (100 μM) in PBS (pH 7.4) were incubated at 37 °C and 30 rpm for 120 h. The samples were diluted to 10 μM with PBS before and after the incubation and subjected to CD measurements in a 1 mm quartz cuvette. Results were corrected by subtracting the values of buffer.

### ATR-FTIR spectrometry

The ATR-FTIR spectra were measured using a Jasco FTIR spectrometer FT/IR-4700 equipped with an ATR PKM-Ge-L reflectance accessory. An aliquot of αS samples (100 μM) in PBS (pH 7.4) was spread on the germanium waveguide and dried under flowing nitrogen gas. ATR-FTIR spectra in the wavenumber range of 1000–3500 cm^−1^ were captured at a resolution of 4 cm^−1^ with 256 accumulations under continuous nitrogen purge. To evaluate the secondary structure of the proteins, the amide I area (1600–1700 cm^−1^) in the spectra was deconvoluted using a Spectra Manager Software (Jasco, Tokyo, Japan).

### TEM

The samples were negatively stained with phosphomolybdic acid solution. The TEM measurements were performed using a JEOL JEM-1200EX transmission microscope (JEOL, Tokyo, Japan) at an acceleration voltage of 80 kV.

### TIRFM

The TIRFM measurements were performed using an inverted microscope (IX70; Olympus, Tokyo, Japan). Excitation of ThT was induced using an argon laser (185F02-ADM; Spectra Physics, Mountain View, CA). Bandpass filtering (D490/30 Omega Optical, Brattleboro, VT) was applied to the fluorescence image before processing using an image intensifier (model VS4-1845; Video Scope International, Sterling, VA) coupled with an SIT camera (C2400-08; Hamamatsu Photonics, Shizuoka, Japan)^83^.

### Statistical analysis

Data were analyzed using unpaired Welch’s t-test or one-way analysis of variance, followed by Dunnett’s multiple comparisons test. All statistical analyses were performed using Prism 8 software (GraphPad Software, La Jolla, CA, USA). Differences were considered significant at *p* < 0.05.

## Supplementary Information

Supplementary Information.

## Data Availability

All data generated or analyzed in this study are included in this article and its Supplementary Information files.
