# Supplementary material for: Intramolecular interaction kinetically regulates fibril formation by human and mouse α-synuclein
Source: Sci Rep. 2023 Jul 5;13:10885. doi: 10.1038/s41598-023-38070-4 (PMC10322983; doi:10.1038/s41598-023-38070-4)
Supplement: Supplementary file 1 — Supplementary Information. [file 41598_2023_38070_MOESM1_ESM.pdf]

Supplementary Information

Intramolecular interaction kinetically regulates fibril formation by human and mouse α-synuclein

Takashi Ohgita[1,*], Hiroki Kono[1], Izumi Morita[2], Hiroyuki Oyama[2], Toshinori Shimanouchi[3], Norihiro Kobayashi[2], Hiroyuki Saito[1]

[1] Laboratory of Biophysical Chemistry, Kyoto Pharmaceutical University, 5 Misasagi-Nakauchi-cho, Yamashina-ku, Kyoto 607-8414, Japan

[2] Department of Bioanalytical Chemistry, Kobe Pharmaceutical University, 4-19-1 Motoyama-Kitamachi, Higashinada-ku, Kobe, 658-8558, Japan

[3] Graduate School of Environmental and Life Science, Okayama University, Okayama 700-8530, Japan

This supplementary data consist of: Tables S1 and S2 and Figures S1–S13

Table S1. Kinetic parameters for fibril formation of human αS (h-αS) and mouse αS (m-αS).

	F_0	F_{max}	k_{app} (h^{-1})	$t_{1/2}$ (h)	Lag time (h)
h-αS WT	0	1276 ± 135	0.07 ± 0.03	79 ± 7	50 ± 13
m-αS WT	0	6980 ± 237	0.6 ± 0.07	7 ± 0.3	4 ± 0.5

The values and standard errors for maximum fluorescence intensity (F_{max}), apparent elongation rate constant (k_{app}), and half time ($t_{1/2}$) for αS fibril formation were determined from the analysis of ThT fluorescence curves using equation 1, as shown in Fig. 2A. The initial fluorescence intensity (F_0) was set to 0 for the analysis. Lag times were calculated as $t_{1/2} - 2/k_{app}$, and the errors were determined from those of k_{app} and $t_{1/2}$ according to the principle of propagation of error [*J. Res. Natl. Bur. Stand.* 1966, 70C, 263–273].

Table S2. Secondary structure components of monomeric or fibrillar h-αS and m-αS.

		Secondary structure (%)		
		Random coil/ α-Helix	β-sheet	Turn
h-αS	Monomer	68.4	27.3	4.2
	Fibrils	43.8	56.2	—
m-αS	Monomer	73.2	20.6	6.2
	Fibrils	53.0	44.3	2.7

Components of the secondary structure were determined using Fourier self-deconvolution of the attenuated total reflection Fourier-transform infrared spectra shown in Fig. 3B. Assignment of the band positions to the secondary structure were referenced from *Prog. Biophys. Mol. Biol.* 1993, 59, 23–56 and *Subcell. Biochem.* 1994, 23, 405–450.

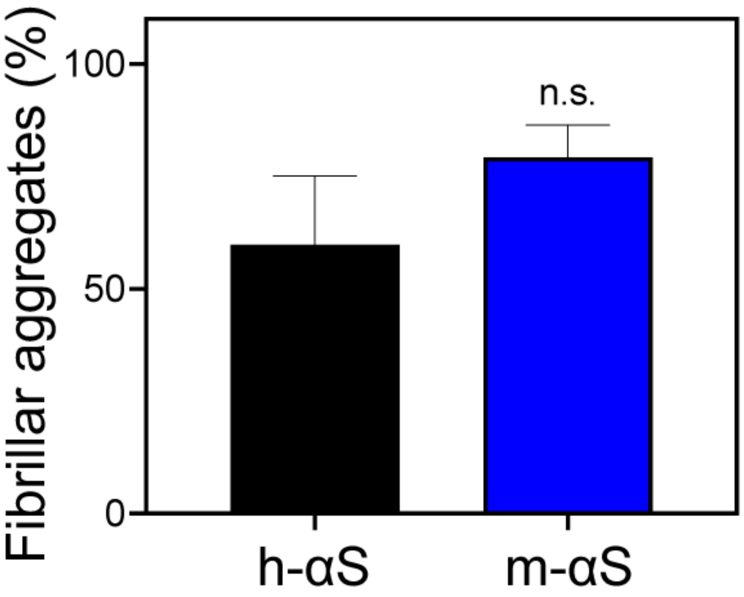

Figure S1. Amounts of fibrils in the αS solution at plateau phase. After incubating the αS solution (100 μM) at 37 °C for five days, insoluble αS fibrils were precipitated as a pellet using ultracentrifugation at 280,000 g for 1.5 h. The pellet was solubilized through overnight incubation with 4 M urea, and the protein concentration was determined using Lowry methods [*Sci. Rep.* 2022, 12, 6770]. The e*rror bars* represent the standard errors. n.s., not significant.

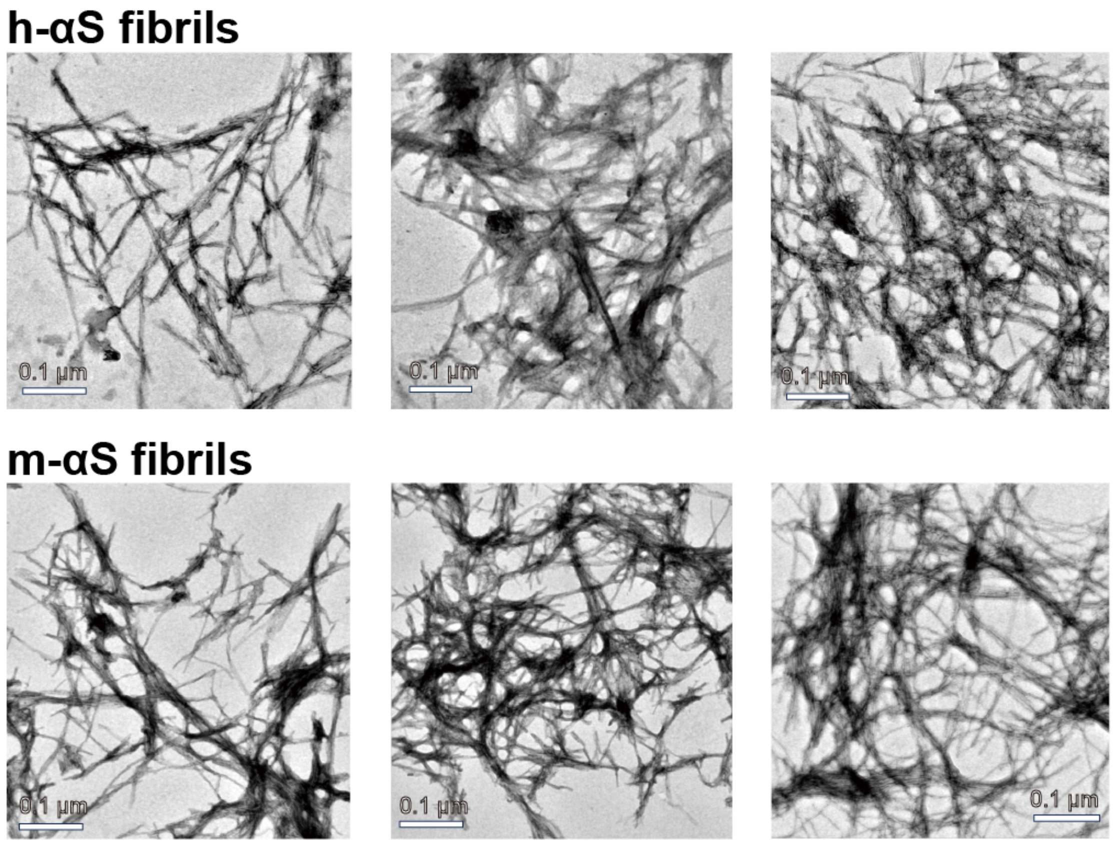

Figure S2. Transmission electron microscopy (TEM) images of h-αS (upper) and m-αS (lower) fibrils at three different fields of view. *Scale bars* represent 0.1 µm.

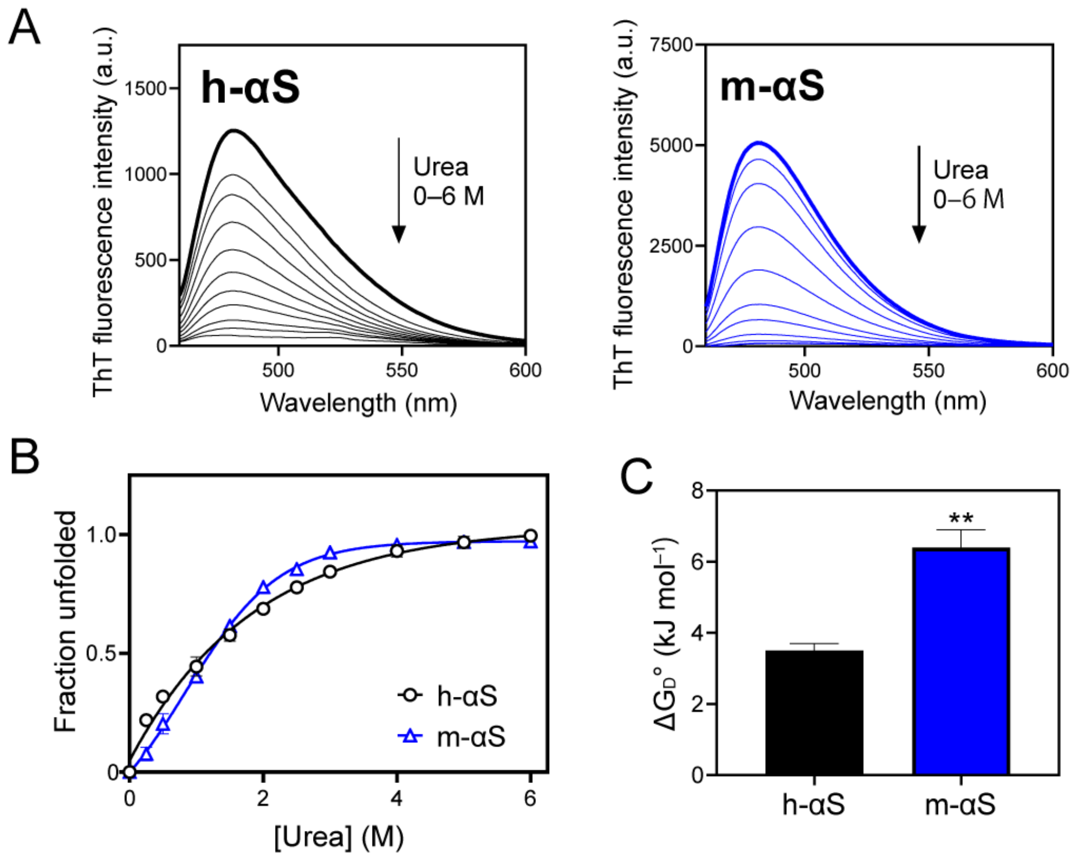

Figure S3. Urea denaturation of h-αS and m-αS fibrils. (A) Changes in ThT fluorescence spectra of h-αS (left panel) and m-αS (right panel) with different concentrations of urea. The fibrils were prepared by incubating 100 μM of αS solutions at 37 °C for five days. (B) Urea-induced denaturation curves of h-αS (black circle) and m-αS (blue triangle) fibrils. (C) Comparison of the change in Gibbs free energy of denaturation ($\Delta G_D°$) of h-αS and m-αS fibrils. The $\Delta G_D°$ values were determined as $\Delta G_D = \Delta G_D° - m[\text{urea}]$, where $\Delta G_D = -RT \ln K_D$. The *error bars* represent the standard errors. $^{**}p < 0.01$ compared with h-αS.

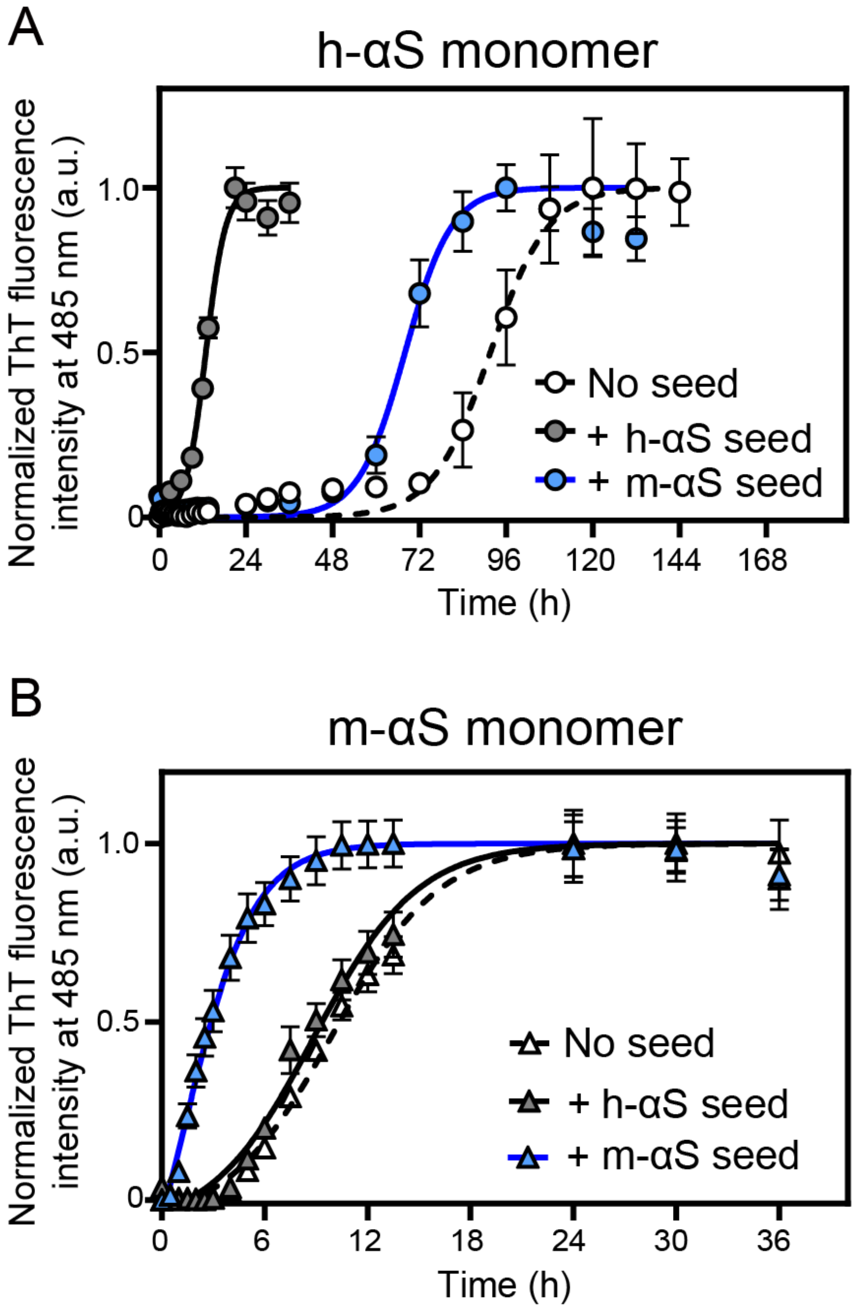

Figure S4. Cross-seeding aggregation between h-αS and m-αS. Traces of ThT fluorescence for fibril formation of 20 μM of h-αS (*A*) and m-αS (*B*) in the presence or absence of 4 μM of preformed h-αS or m-αS seed fibrils. The experiments were independently repeated three times, and five samples were used for each replicate. The *solid* and *dotted lines* represent the curves fitted using the sigmoidal equation (1). *Error bars* represent S.E. *a.u.*, arbitrary units.

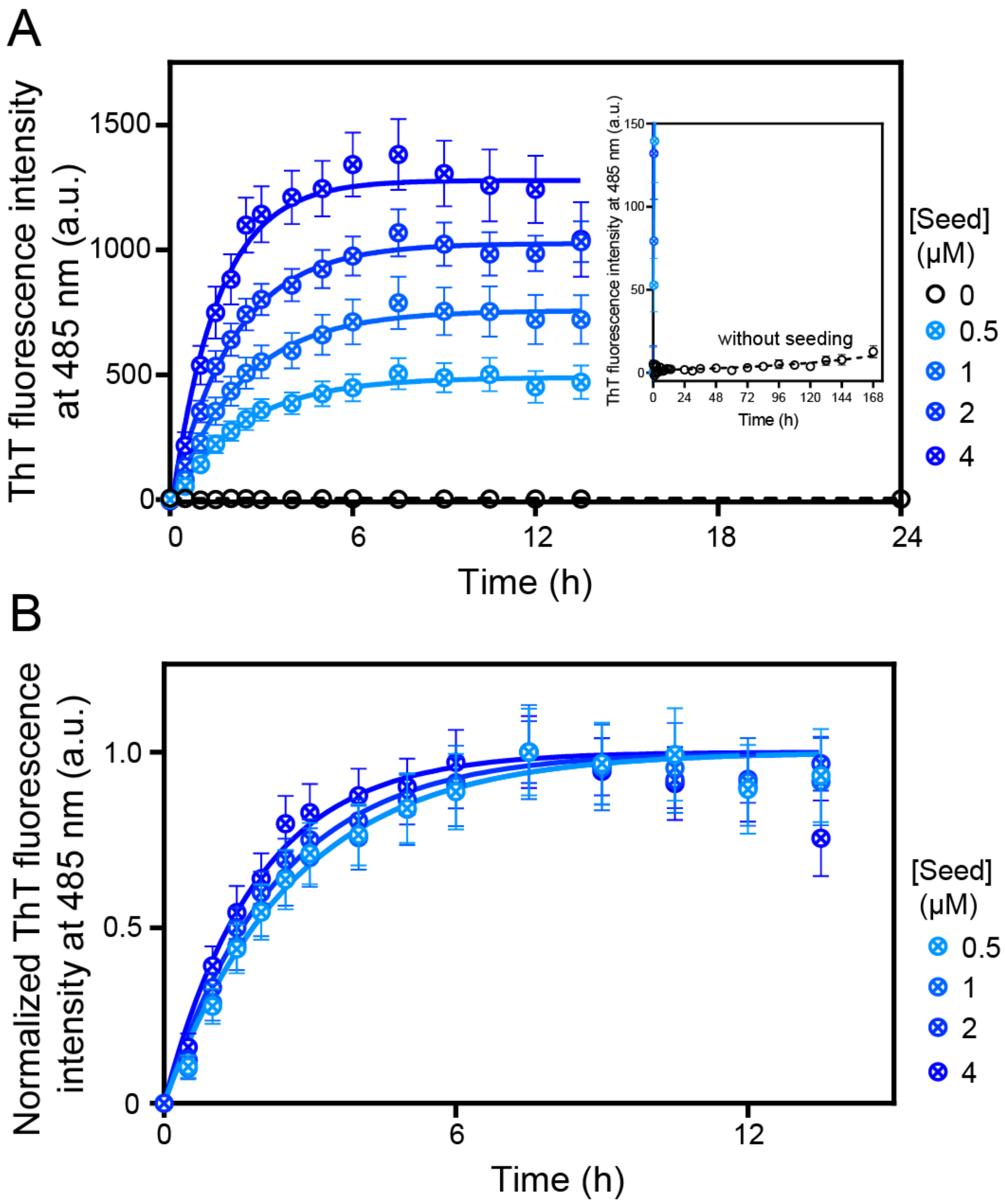

Figure S5. Fibril formation of m-αS with varying concentrations of seed fibrils under quiescent conditions. Traces of ThT fluorescence (*A) or* normalized ThT fluorescence curves (*B*) for fibril formation of 20 μM of m-αS with various concentrations of preformed seed fibrils. The inset shows the change in the ThT fluorescence of m-αS without seeding. The experiment was independently repeated three times, and five samples were used for each replicate. *Error bars* represent S.E. *a.u.*, arbitrary units.

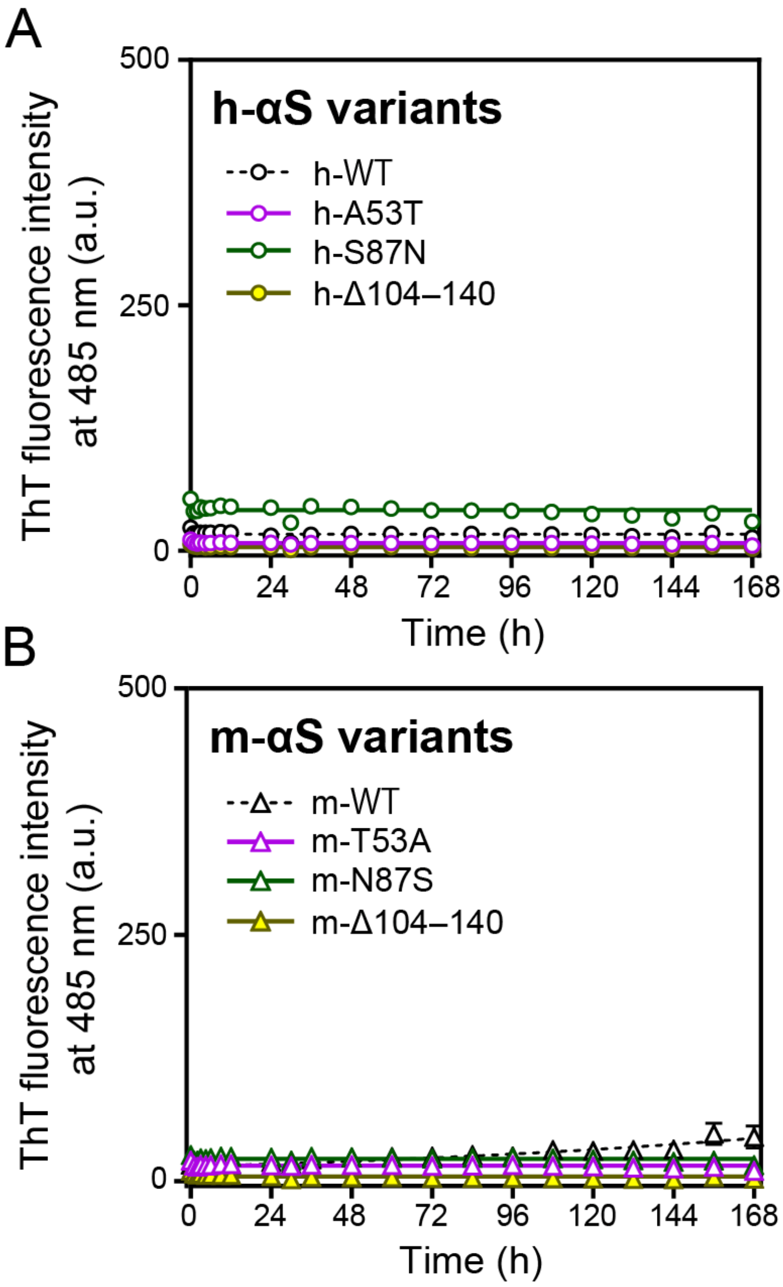

Figure S6. ThT fluorescence assay for fibril formation of h-αS, m-αS, and their variants under quiescent conditions. Traces of ThT fluorescence for fibril formation of 50 μM of WT and the variants of h-αS (*A*) and m-αS (*B*). The experiments were independently repeated three times, and five samples were used for each replicate. *Error bars* represent S.E. *a.u.*, arbitrary units.

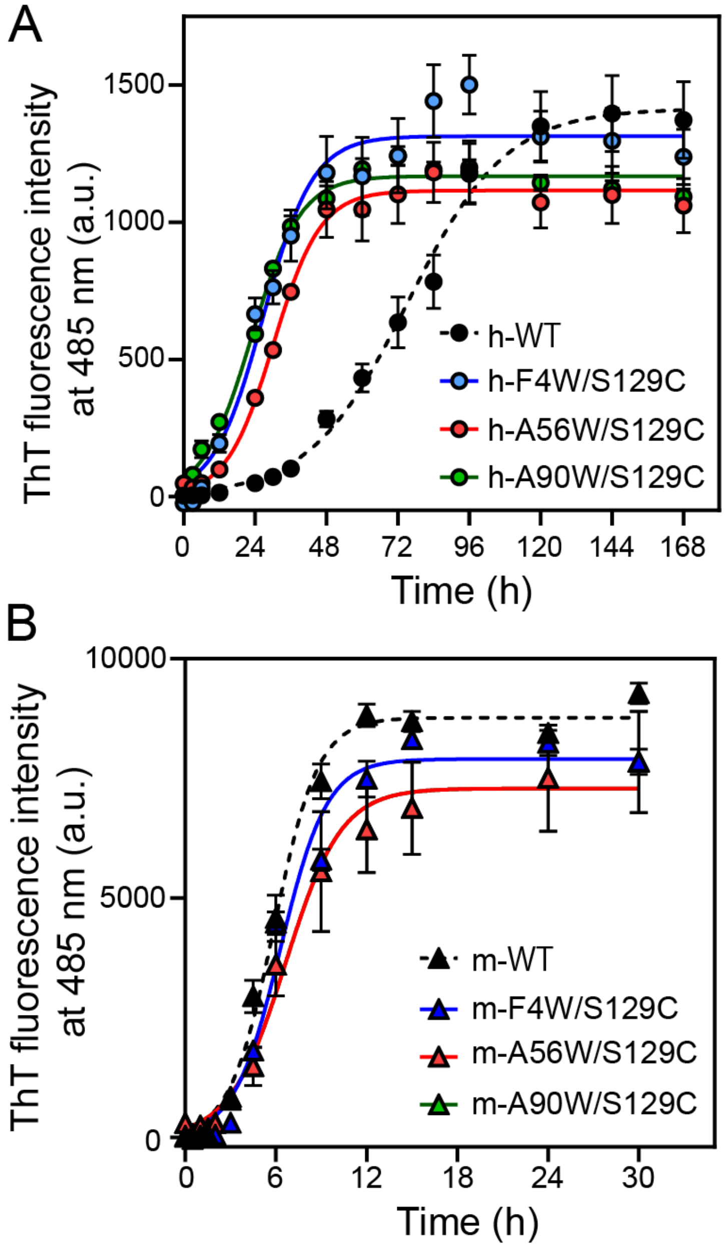

Figure S7. Traces of thioflavin T fluorescence of the h-αS and m-αS Trp/Cys variants. ThT fluorescence curves of 50 μM of the Trp/Cys variants of h-αS (*A*) and m-αS (*B*). WT (black), F4W/S129C (blue), A56W/S129C (red), and A90W/S129C (green). The experiments were independently repeated three times, and five samples were used for each replicate. The *solid* and *dotted lines* represent the curves fitted using the sigmoidal equation (1). *Error bars* represent S.E. *a.u.*, arbitrary units.

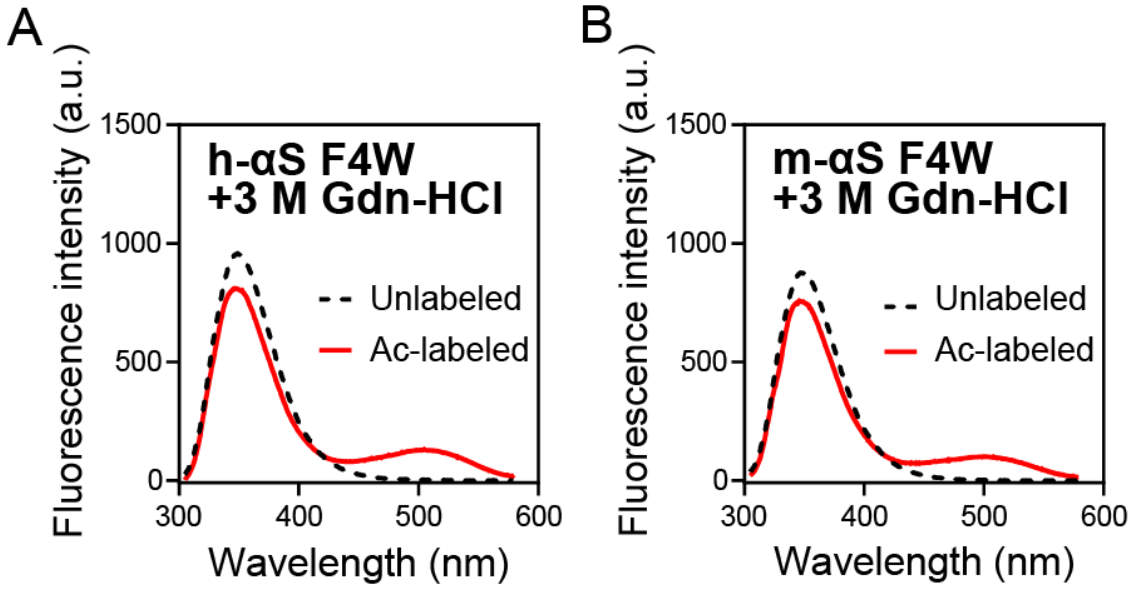

Figure S8. Fluorescence emission spectra for the unlabeled and Ac-labeled F4W/S129C variants of h-αS (*A*) and m-αS (*B*) with 3 M guanidine hydrochloride (Gdn-HCl). The protein concentration was 2 μM. The measurements were repeated thrice.

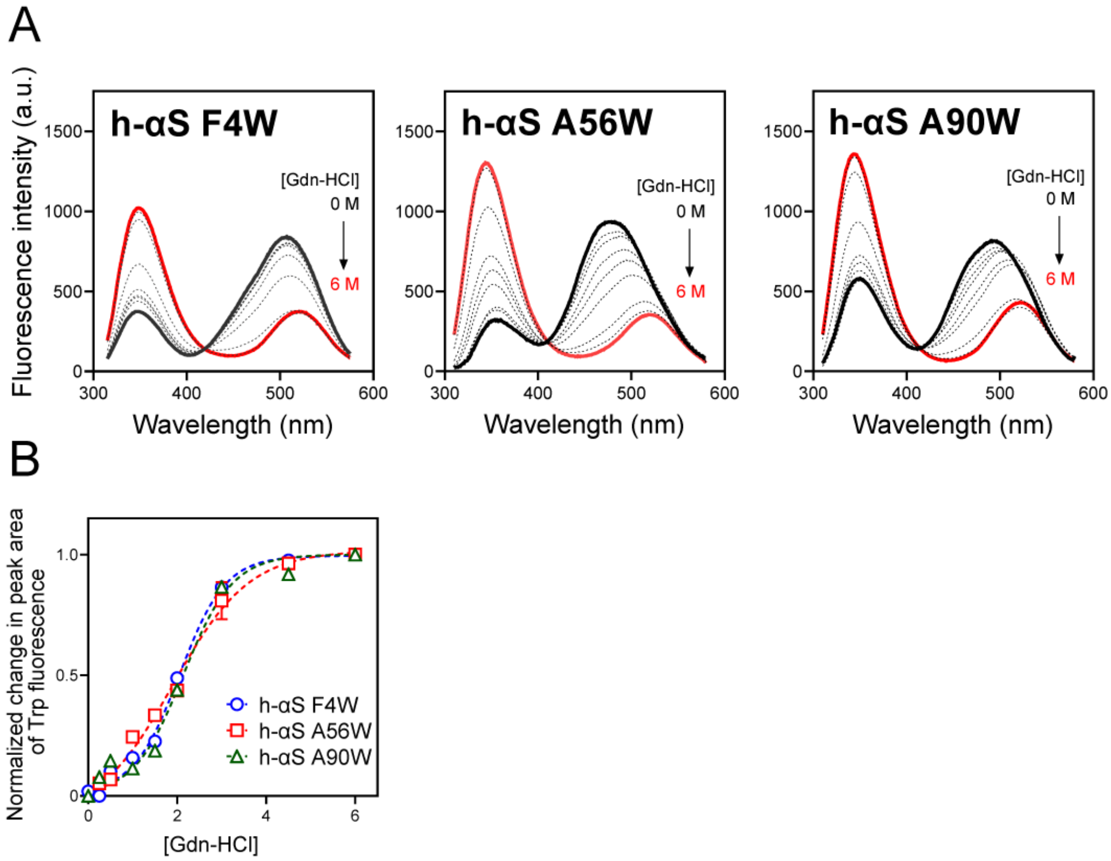

Figure S9. Changes in the fluorescence spectra of the Ac-labelled h-αS F4W/S129C, A56W/S129C, or A90W/S129C variants with increasing concentrations of guanidine hydrochloride (Gdn-HCl). The protein concentration was 2 μM. The measurements were repeated thrice.

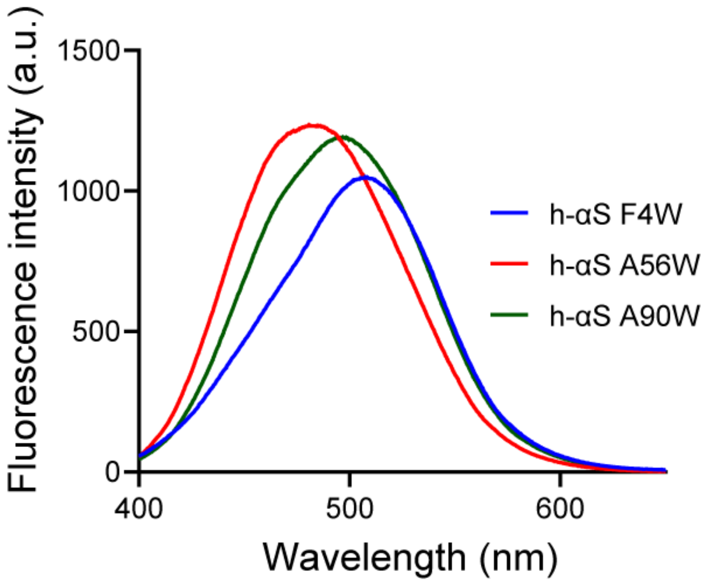

Figure S10. Emission spectra of the Ac-labelled h-αS F4W/S129C, A56W/S129C, or A90W/S129C variants with excitation wavelength of 340 nm. The protein concentration was 2 μM. The measurements were repeated thrice.

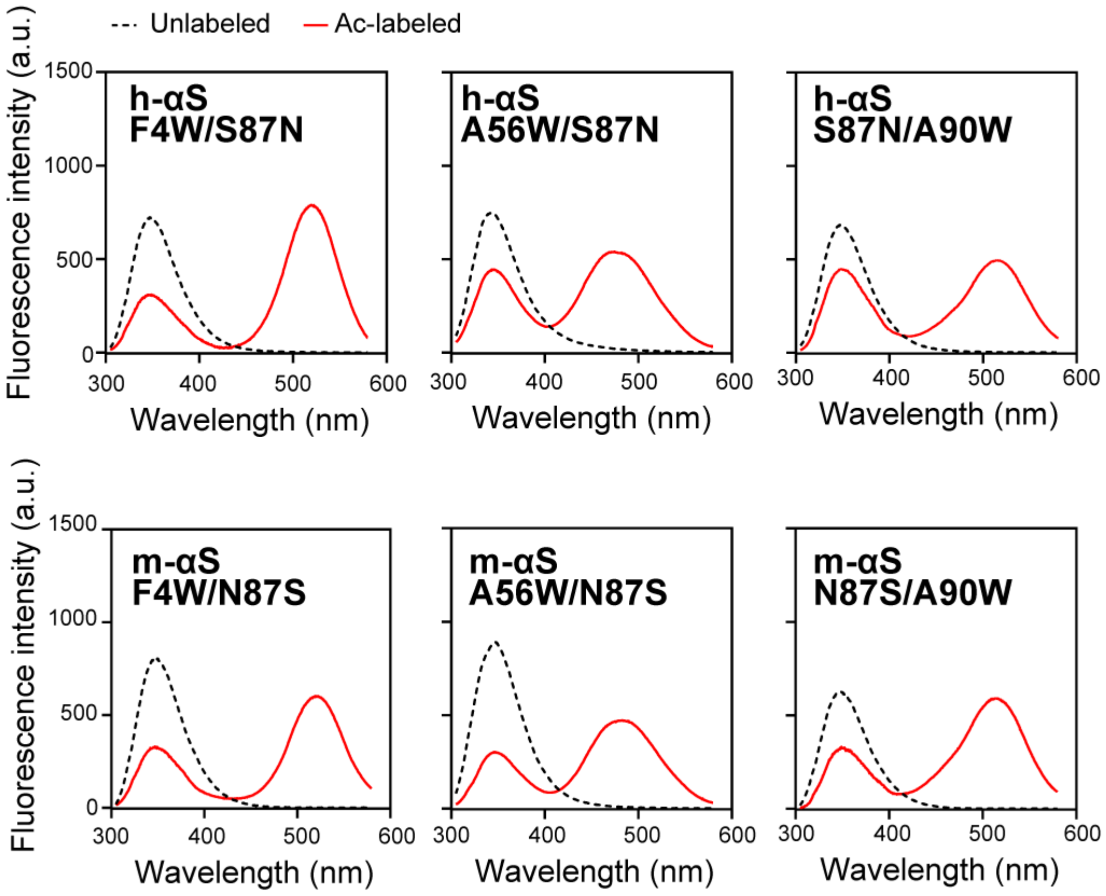

Figure S11. Fluorescence emission spectra for the unlabeled and Ac-labeled h-αS S87N (upper) and m-αS N87S (lower) variants that have Trp residue at different sites. The protein concentration was 2 μM. The measurements were repeated thrice.

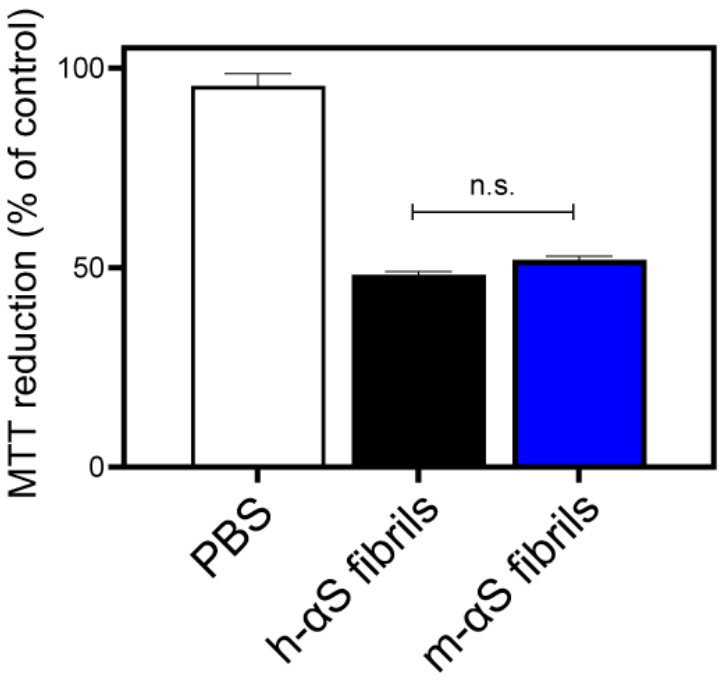

Figure S12. Cytotoxicity of the h-αS and m-αS fibrils. h-αS and m-αS fibrils were prepared by incubating 100 μM of αS solution at 37 °C for five days. CHO-K1 cells were plated at a density of 1×10^4 cells/well on 24-well plates in Dulbecco's modified Eagle's medium (DMEM) containing 2% fetal bovine serum (FBS). After incubation for 24 h, 1 μM of αS fibrils in phosphate-buffered saline (PBS) was added. The cells were further incubated with DMEM containing 1% FBS for 24 h. After the addition of 3-(4,5-dimethylthiazol-2-yl)-2,5-diphenyltetrazolium bromide (MTT) at a final concentration of 0.5 mg/mL, the cells were further incubated for 2 h. The culture media were then removed and replaced with dimethyl sulfoxide to dissolve the resultant formazan crystals. Absorbance of formazan was measured at 555 nm, with absorbance subtraction at 750 nm. The *error bars* represent the standard errors. n.s., not significant.

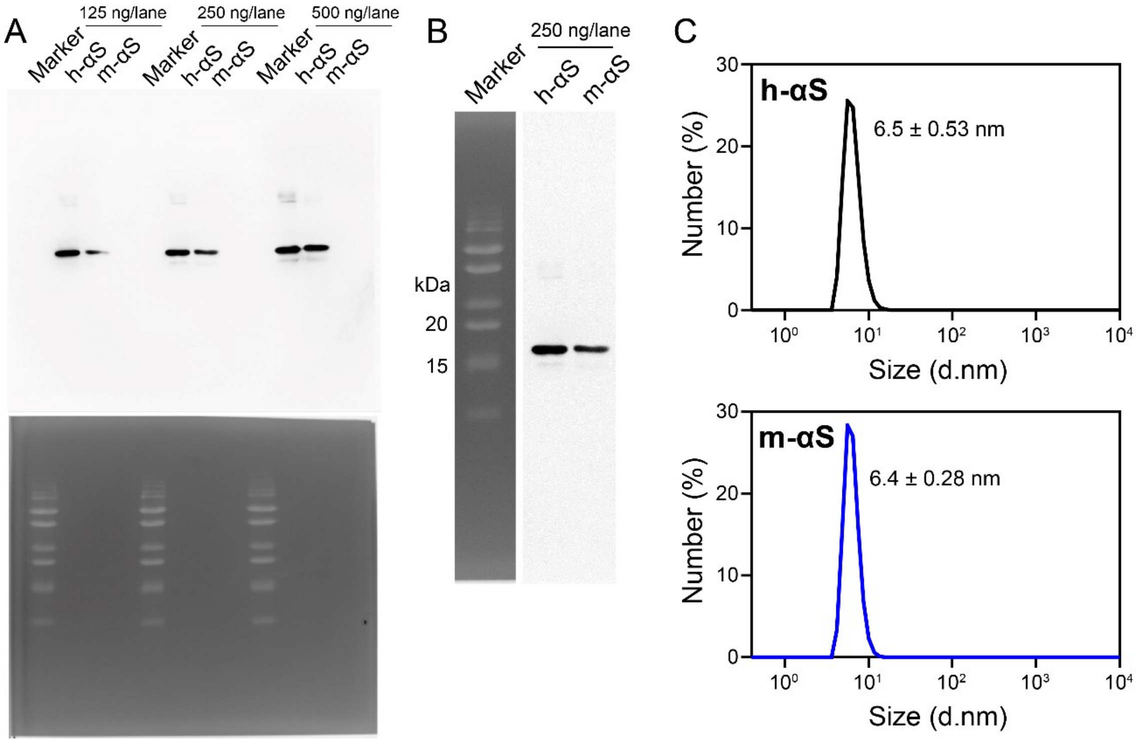

Figure S13. Purity of the initial αS monomer solutions used in ThT fluorescence assay. *A* and *B*, Full (*A*) and cropped (*B*) western blotting images of the initial monomer solutions of the h-αS and m-αS just after the refolding procedure. *A*, Chemiluminescent (*upper*) and brightfield (*lower*) images. The h-αS and m-αS solutions (125, 250, or 500 ng of protein/lane) were subjected to SDS-PAGE with 15% gels and transferred to polyvinylidene difluoride membranes. After the blocking, the membranes were probed with the anti-αS polyclonal antibody (Proteintech, #10842-1-AP), followed by the horseradish peroxidase-conjugated AffiniPure goat anti-rabbit IgG antibody (Jackson ImmunoResearch, Inc., West Grove, PA) and the ECL Prime Western Blotting Detection Reagent (GE Healthcare, Milwaukee, WI). *C*, The particle size distribution in the initial monomer solutions of the h-αS (*upper*) and m-αS (*lower*) determined by the dynamic light scattering measurements. The measurements were performed three times using independently prepared samples. The values in the graph represent the peak sizes (average ± standard error).
